# Chemical complexity of odors increases reliability of olfactory threshold testing

**DOI:** 10.1038/srep39977

**Published:** 2017-01-10

**Authors:** Anna Oleszkiewicz, Robert Pellegrino, Katharina Pusch, Celine Margot, Thomas Hummel

**Affiliations:** 1 Interdisciplinary Center, “Smell & Taste”, Department of Otorhinolaryngology, TU Dresden, Germany; 2 Institute of Psychology, University of Wroclaw, Poland

## Abstract

Assessment of odor thresholds is a widely recognized method of measuring olfactory abilities in humans. To date no attempts have been made to assess whether chemical complexity of odors used can produce more reliable results. To this end, we performed two studies of repeated measures design with 121 healthy volunteers (age 19–62 years). In Study 1, we compared thresholds obtained from tests based on one odor presented in a pen-like odor dispensing device with three odors and six odors mixtures presented in glass containers. In study 2 we compared stimuli of one and three odors, both presented in glass containers. In both studies measurements were performed twice, separated by at least three days. Results indicate that the multiple odor mixtures produced more reliable threshold scores, as compared to thresholds based on a single substance.

There are multiple methods designed to assess olfactory function in humans^1^, with the Sniffin’ Sticks (SnSt)^2^,^3^ being considered as one of the most popular. The SnSt consists of three different tests - olfactory threshold, odor discrimination and odor identification. It enables a detailed diagnosis of olfactory impairments^4^.

Odor thresholds are typically assessed for single substance, e.g. n-butanol or phenyl ethyl alcohol (PEA)^5^. This is interesting as results from these measurements – despite of relatively large variation – correlate with the overall chemosensory sensitivity of the tested person although it can be assumed, that the single substance only activates a certain portion of olfactory receptors. In this context it is important to know that humans differ in the expression of olfactory receptors^6^,^7^. Thus, it would appear to be logical to investigate thresholds for several odors or mixtures of odors. However, the question of the chemical complexity of odors used in olfactory testing has rarely been studied^8^,^9^ and up-to-date results can be considered as inconclusive.

Human ability to recognize a wide variety of odorants is the result of the high number of olfactory receptors^10^,^11^ encoded by 339 intact genes^12^. The process of detecting odors starts with olfactory receptors, located in the cilia of the olfactory sensory neurons^13^. Odorant molecules bind to these relatively unspecific receptors which may elicit a cellular response, which may be transmitted to the olfactory bulb. Next, the signals are transmitted further to the olfactory cortex^10^,^14^,^15^. Olfactory receptors can accept a range of odor molecules, and a substance containing single type of odorant molecules may bind to various types of olfactory receptors^10^,^16^,^17^,^18^. Therefore, it can be assumed that a mixture of multiple odors (containing various molecules) can activate more receptors at once than a single-type odorous molecules.

The aim of the current work was to test whether chemically more complex stimuli can provide more reliable results than examination with single-type molecules. Because of variability in receptor expression across the population^19^,^20^, results based on a mixture of odors can be expected to be more reliable than those related to single-type molecules. To this end, we performed two studies where we controlled for the method of presentation (odor presentation with the use of the SnSt pen or in a glass container) and the number of odors used for stimulation (one odor, three odors mixture or six odor mixture).

## Results

### Study 1

**Figure 1:**
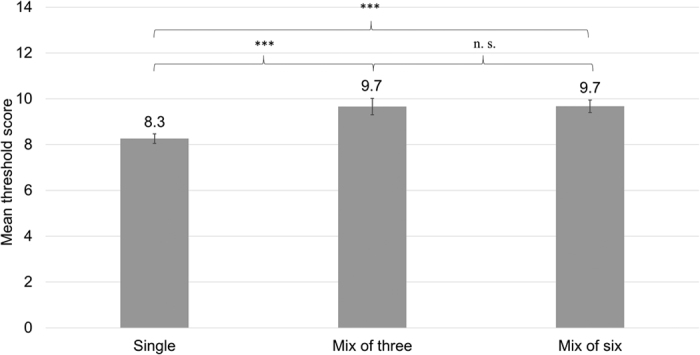
Mean olfactory threshold scores depending on the number of odors in the mixture presented to the subject.

We conducted the linear mixed model (LMM) with maximum-likelihood estimation. Within the model we included the number of odors in the mixture (single, mix of 3 and mix of 6 odors), the number of session and subjects’ sex as fixed factors. The type of multiple odors mixture and sequence of threshold tests were treated as a random factor. Within the tested model we found significant main effect of the number of odorants, *F*(2, 520) = 13.2, *p* <* *0.001 (Fig. 1). Pairwise comparisons revealed that threshold results obtained with the tests using one odor were significantly lower (*M* = 8.3 ± 0.2) than thresholds obtained with odor mixtures of three odors (*M* = 9.7 ± 0.4; *p *=* *0.001) or six odors (*M* = 9.7 ± 0.3; *p < *0.001). There was no significant difference between thresholds in three and six odor conditions (*p* =* *0.95; see Table 1). We also found the main effect of subjects’ sex, *F*(1,478) = 7.7, *p* =* *0.006, indicating that in general women (*M* = 9.5 ± 0.5) performed better than men (*M* = 8.8 ± 0.6). There were no other significant main or interaction effects (all *F*s < 2.1, *p*s >* *0.12).

Additionally, we checked the test-retest reliability of threshold measures obtained during the two sessions, indicated by Pearson’s *r*. We found that reliability of the test based on one odor given in the SnSt pen was *r* =* *0.31, *p* =* *0.003, whereas for the three odors mixture given in a glass container it was *r* =* *0.57, *p* <* *0.001 and for the six odors mixture it was *r* =* *0.56, *p* <* *0.001.

### Study 2

We tested the linear mixed model (LMM) with maximum-likelihood estimation. Within the model the number of odors in the mixture of stimuli (single and mix of three odors), the number of session and subjects’ sex were used as fixed factors. The type of multiple odor mixture was treated as a random factor. Data revealed a main effect of subjects’ sex, *F*(1, 29) = 9.7, *p* =* *0.004, indicating that females performed significantly better (*M* = 9.2 ± 0.4) than their male counterparts (*M* = 7.7 ± 0.4). We also found a main effect of the number of odors, *F*(1,87) = 73.1, *p* <* *0.001, indicating, that testing with the mixture of three odors resulted in significantly higher threshold scores (*M* = 10.1 ± 0.3), as compared to the test based on a single odor (*M* = 6.8 ± 0.3; see Table 1). No other main or interaction effects were significant (all *F*s < 1.9; *p*s* > *0.05).

The test-retest reliability analysis showed that the test based on a single odor was less reliable, *r* =* *0.20, *p* =* *0.27, than the test based on the mixture of three odors, *r* =* *0.52, *p* =* *0.003.

Descriptive statistics for thresholds produced with single odor, mix of three odors (three variants) and mix of six odors for both studies can be found in Table 2.

## Discussion

With the two experiments we offer empirical proof for higher reliability of tests involving odor mixtures, as compared to tests based on a single odor, and for more favorable treatment of the complex odor stimuli. In the first experiment, the test-retest reliability of the test based on a single odor stimulus was relatively low^21^, as opposed to both tests based on multiple odor mixture stimuli. A similar pattern of results was observed in the second experiment. Although test-retest reliability of the test based on the mixture of three odors did not reach the conventional level of 0.70, it was still two times higher than reliability of the single-odor test that did not reach the significance level. Observed higher reliability and more favorable treatment of the subjects in multiple odor tests might result from more varied molecules included in the odor mixture that activate more receptors. Therefore, the use of odor mixtures might be more resistant to variability in the individual olfactory receptors expression across the population^6^,^19^,^20^. This finding contradicts former reports on comparable reliability of tests using odors mixtures and their components^9^. We assume that this difference might result from the fact, that in the mentioned study researchers engaged subjects in five sessions, what could result in observed improvement in performance across sessions^22^, which was not the case in the current study.

We also found significant sex-related differences in both studies, indicating that women performed better than men and that this effect was independent from the type of stimulus. This finding is convergent with former reports, showing that generally women have higher olfactory sensitivity and obtain better results in olfactory tests than men^23^,^24^,^25^. With the current study we supplemented this finding by showing that females outperform their male counterparts also in olfactory tasks involving multiple odor stimuli.

The fact that we repeatedly tested threshold of individual subjects can be considered as a potential limitation of the study. Threshold testing is demanding and tiring. Therefore, subjects in our studies might have felt exhausted after the first session. Nevertheless, statistical analyses revealed no significant decrease in results obtained in the first session, as compared to the second attempt. Further studies could potentially verify, whether the effect of decreased and more stable threshold results can be observed in subjects given more time to rest between the sessions.

Limitations of the present work might also relate to the fact that investigated populations did not involve individuals with olfactory loss what potentially limits the comparability between the presently obtained results and established clinical tests. Future studies could verify whether threshold obtained with the proposed approach coincide with clinical tests results.

To sum up, threshold test based on varied odors produces more stable and reliable within-subject scores compared to the presentation of single-type molecules. This is assumed to be due to the more efficient activation of olfactory receptors.

## Methods

### Ethics statement

The study was performed in accordance to the Declaration of Helsinki on Biomedical Studies Involving Human Subjects. Informed written consent was obtained from all the participants. The study design and consent approach was approved by the University of Dresden Medical Faculty Ethics Review Board (EK6702010).

### Participants

In the first study participated 90 healthy subjects, aged from 19 to 34 years (*M* = 23.6, *SD* = 2.4). Of those, 72 were females between 19–34 years (*M* = 23.6, *SD* = 2.3) and 18 were males between 19–29 years (*M* = 23.5, *SD* = 2.6). In the second study participated 31 healthy subjects, aged from 19 to 34 years (*M* = 23.6, *SD* = 2.4). Of those, 16 were females between 20–62 years (*M* = 30.6, *SD* = 10) and 15 were males between 21–55 years (*M* = 23.6, *SD* = 9.1). All studies were conducted at the Department of Otorhinolaryngology of the “Technische Universität Dresden”. The participants constituted a sample of convenience. Statistical analyses was performed with SPSS v. 21 (SPSS Inc., Chicago, IL, USA) with *p* < 0.05 set as the level of significance.

### Stimuli

In the first study, three number-of-odors conditions were designed to measure threshold: a mixture of six odors (both presented in brown glass bottles of 60 ml volume, height 65 mm, diameter of opening 35 mm); three mixtures of three odors (presented in a bottle); and butanol (presented infelt-tip pens typical of Sniffin’ Sticks’). Due to the multitude of distinguishable odor qualities, we decided to use the framework of Henning’s ‘smell prism’ to select six odors representing primary odor categories: *flowery* (e.g., rose), *foul; fruity* (e.g., lemon), *spicy* (e.g., cloves), *burnt*; and *resinous* (e.g., eucalyptus)^26^,^27^,^28^,^29^. The three odor mixtures represented smaller variants of these odors (see: Table 3). The six odors that were used: Geraniol, Anethol, Tanol, Cineol, Citronellal and Isobutyraldehyd. The three variants of the initial six odors were used in three odor mixtures: A) Cineol, Tanol, Anethol, B) Geraniol, Cineol, Isobutyraldehyd C) Anethol, Citronellal, Cineol.

The second study was designed to present another single odor (phenylethyl alcohol, PEA) and two mixtures using the same dispensing bottles as in the first study. The two mixtures used were D) Anethol, Geraniol, Citronellal, C) Anethol, Citronellal, Cineol.

Intensity of all mixtures’ components was assessed in a preliminary study (n = 10) where individuals rated the intensity and hedonics using a ten-point Likert-type scale. No differences between intensities were observed (*p* <* *0.05), thus the isointensity of the mixtures’ components was assumed.

### Procedure

Participants of both studies were first reviewed with a questionnaire to determine any medication or past history that could potentially influence their olfactory abilities. Less than 10% of the sample reported regular smoking. Each individual was assessed on their ability to identify smells at a supra-threshold level with the identification subtest from the Sniffin’ Sticks’ battery test^2^. All participants were asked to not smoke, eat or drink anything other than water for approximately 30 minutes prior to all tests procedures. Additionally, individuals were asked to refrain from using a strong perfumes or fragrances on the day of testing.

For all number-of-odors conditions, the threshold was surveyed in a triple-forced choice paradigm where participants had to discriminate the odor from two blanks (filled with solvent propylene glycol). Odor and blanks were placed about 2 cm in front of both nostrils of the participant for 3 seconds. Beginning with the lowest odor concentration, a staircase paradigm was used where two correct or one incorrect answer resulted in a decrease or increase of concentration, a so-called turning point. The threshold score was the mean of the last four turning points in the staircase. The highest concentration was 4% odor solution (diluted with propylene glycol) while the subsequent concentrations were further diluted (1:2 fashion) to create 16 concentrations. For both bottles and pens, 3 mL of the odor and blanks solutions were added. In both studies, sessions were separated by several days (*M* = 5.07, *SD* = 3.85) with minimum time of 24 hours. In study 1 three thresholds were tested per day; in study 2 it was two thresholds per day. Within each session, there were 15 minutes break between the threshold tests.

## Additional Information

**How to cite this article**: Oleszkiewicz, A. *et al*. Chemical complexity of odors increases reliability of olfactory threshold testing. *Sci. Rep.* **7**, 39977; doi: 10.1038/srep39977 (2017).

**Publisher's note:** Springer Nature remains neutral with regard to jurisdictional claims in published maps and institutional affiliations.

## Figures and Tables

**Table 1:** Mean threshold scores for male and female subjects across the two sessions (values in brackets represent standard deviation).

	session 1		session 2	
	males	females	males	females
single odor	7.8 (2)	8.2 (1.6)	8.6 (2.4)	8.5 (2)
mix of 3 odors	9.7 (2.6)	9.7 (2.8)	9 (2.7)	10.1 (3)
mix od 6 odors	9.1 (2.4)	10.4 (2.4)	8.7 (2.2)	10.1 (2.1)

**Table 2:** Descriptive statistics for thresholds produced with single odor, mix of three odors (three variants) and mix of six odors (A) Cineol, Tanol, Anethol, B) Geraniol, Cineol, Isobutyraldehyd C) Anethol, Citronellal, Cineol, D) Anethol, Geraniol, Citronellal).

Study 1				
	*M*	*SD*	Min	Max
Session 1				
Six odors	10.1	2.4	4.3	15.8
Three odors (A)	8.3	2.8	4.5	15.5
Three odors (B)	11.1	2.3	7.5	16.0
Three odors (C)	9.7	2.5	5.3	14.5
Single odor	8.1	1.7	4.0	13.0
Session 2				
Six odors	9.8	2.2	3.8	16.0
Three odors (A)	8.0	2.7	3.5	15.5
Three odors (B)	11.5	2.7	8.5	16.0
Three odors (C)	10.2	2.7	4.5	14.3
Single odor	8.5	2.1	4.0	15.3
Study 2				
	*M*	*SD*	Min	Max
Session 1				
Three odors (D)	10.4	2.9	5.5	15.3
Three odors (C)	10.1	2.3	6.8	13.8
Single odor	6.4	2.5	2.0	15.3
Session 2				
Three odors (D)	9.8	2.4	6.5	15.5
Three odors (C)	10.3	2.2	7.3	15.5
Single odor	7.2	2.5	4.8	15.5

**Table 3:** Characteristics of odors used in the studies.

Odor	Name	Category	Concentration
1	Anethol	Spicy	100%
2	Geraniol	Flowery	14, 30%
3	Tanol	Resinous	16, 78%
4	Cineol	Burnt	5, 00%
5	Citronellal	Fruity	58, 89%
6	Isobutyraldehyd	Foul	6, 25%
